# Synergistically Induced Hypothermia and Enhanced Neuroprotection by Pharmacological and Physical Approaches in Stroke

**DOI:** 10.14336/AD.2017.0817

**Published:** 2018-08-01

**Authors:** Jun Zhang, Kaiyin Liu, Omar Elmadhoun, Xunming Ji, Yunxia Duan, Jingfei Shi, Xiaoduo He, Xiangrong Liu, Di Wu, Ruiwen Che, Xiaokun Geng, Yuchuan Ding

**Affiliations:** ^1^China-America Institute of Neuroscience, Xuanwu Hospital, Capital Medical University, Beijing, China; ^2^Department of Neurosurgery, Wayne State University School of Medicine, Detroit, MI 48201, USA; ^3^China-America Institute of Neuroscience, Luhe Hospital, Capital Medical University, Beijing, China

**Keywords:** Ischemia/reperfusion, combination therapy, apoptosis, ATP, reactive oxygen species

## Abstract

Hypothermia is considered as a promising neuroprotective treatment for ischemic stroke but with many limitations. To expand its clinical relevance, this study evaluated the combination of physical (ice pad) and pharmacological [transient receptor potential vanilloid channel 1 (TRPV1) receptor agonist, dihydrocapsaicin (DHC)] approaches for faster cooling and stronger neuroprotection. A total of 144 male Sprague Dawley rats were randomized to 7 groups: sham (n=16), stroke only (n=24), stroke with physical hypothermia at 31ºC for 3 h after the onset of reperfusion (n=24), high-dose DHC (H-DHC)(1.5 mg/kg, n=24), low-dose DHC (L-DHC)(0.5 mg/kg, n=32) with (n=8) or without (n=24) external body temperature control at ~38 ºC (L-DHC, 38 ºC), and combination therapy (L-DHC+ ice pad, n=24). Rats were subjected to middle cerebral artery occlusion (MCAO) for 2 h. Infarct volume, neurological deficits and apoptotic cell death were determined at 24 h after reperfusion. Expression of pro- and anti-apoptotic proteins was evaluated by Western blot. ATP and reactive oxygen species (ROS) were detected by biochemical assays at 6 and 24 h after reperfusion. Combination therapy of L-DHC and ice pad significantly improved every measured outcome compared to monotherapies. Combination therapy achieved hypothermia faster by 28.6% than ice pad, 350% than L-DHC and 200% than H-DHC alone. Combination therapy reduced (*p*<0.05) neurological deficits by 63% vs. 26% with L-DHC. No effect was observed when using ice pad or H-DHC alone. L-DHC and ice pad combination improved brain oxidative metabolism by reducing (*p*<0.05) ROS at 6 and 24 h after reperfusion and increasing ATP levels by 42.9% compared to 25% elevation with L-DHC alone. Finally, combination therapy decreased apoptotic cell death by 48.5% vs. 24.9% with L-DHC, associated with increased anti-apoptotic protein and reduced pro-apoptotic protein levels (*p*<0.001). Our study has demonstrated that combining physical and pharmacological hypothermia is a promising therapeutic approach in ischemic stroke, and warrants further translational investigations.

Stroke ranks among the most debilitating vascular diseases worldwide resulting in devastating functional impairment and mortality [1]. Every year approximately 795,000 people in the United States experience a stroke killing one person nearly every 4 minutes [2]. In China, the incidence and burden of stroke is increasing rapidly with more than 7 million people suffering from this disease and ~2 million cases being diagnosed each year [3, 4]. Most stroke patients have not benefited from recombinant tissue plasminogen activator (rt-PA), the only drug approved by FDA for ischemic stroke. This is due to rt-PA’s many contraindications, life-threatening complications, and narrow therapeutic window [5].

Therapeutic hypothermia (TH) at mild to moderate levels of body temperature (30-34°C) has long been considered a promising neuroprotective treatment for ischemic stroke [5, 6]. This is evidenced by TH’s ability to reduce oxygen demand, preserve energy stores, and enhance cellular survival [7, 8]. However, clinical applications are still limited given the complications associated with hypothermia, lengthy and labor-intensive nature of administration as well as the need to initiate brain cooling rapidly [9, 10]. Therefore, the search for adjunct therapies is warranted to widen the scope of TH in the clinical setting.

As an alternative to traditional physical therapeutic hypothermia, pharmacological hypothermia (PH) is gaining increasing interest as a possible neuroprotective treatment. Currently, there are 8 classes of drugs being studied in animal models as potential PH agents including Transient Receptor Potential Vanilloid channel 1 (TRPV1) agonists [11]. TRPV1 is a non-specific cation channel thought to provide neuroprotection possibly through its ability to induce hypothermia in rat models [12, 13]. Its agonist, dihydrocapsaicin (DHC), is subsequently being investigated as a promising PH inducer. However, given the toxicity and complications associated with high doses of DHC that are required to achieve effective hypothermia, its sole use as monotherapy is still limited [6].

In the present study, we hypothesized that combining pharmacological and physical approaches provides enhanced hypothermia and neuroprotection. Using the combination therapy, effective hypothermia could be achieved by simultaneously affecting internal thermoregulation (via DHC) and inducing peripheral heat loss (via ice pad). As such, lower doses of treatments and thus fewer complications can be achieved. It is known that during periods of cerebral ischemia, pro-apoptotic proteins such as Caspase-3, Bax become up-regulated leading to neuronal breakup [14-16]. Conversely, pro-survival proteins including Bcl-2 and Bcl-xL play a critical role in cellular survival that function as repressors of cell death [17, 18]. The effects of combination therapy on expression of both protein families were determined. In addition, the effect of treatments on the extent of brain injury, brain ATP and reactive oxygen species (ROS) were assessed. We have shown that combination therapeutic hypothermia achieved with physical (ice pad) and pharmacological (DHC) cooling synergistically induced a better neuroprotection than each alone.

## MATERIALS AND METHODS

### Subject

A total of 144 adult, male Sprague-Dawley rats (300-340 grams, aged at 9 to 10 weeks, from Vital River Laboratory Animal Technology Co Ltd, China) were randomly divided into sham and six MCAO groups with different treatments. The experimental design and procedures were approved by the Institutional Animal Investigation Committee of Xuanwu Hospital at Capital Medical University (China) and were in accordance with the National Institutes of Health (USA) guidelines for care and use of laboratory animals. Sham groups underwent the entire surgical procedure except for embolization (n=16). Six groups with middle cerebral artery occlusion (MCAO) (n=24 per group) were used: 1) normothermia (rectal temperature at 37.8-38.3°C) with saline injection, 2,3) L-DHC (0.5 mg/kg) at 31°C or at 37.8 - 38.3°C, 4) H-DHC (1.5mg/kg, at 31°C), 5) physical hypothermia (ice pad, at 31°C), 6) L-DHC+ice pad (31°C). Treatments were administered at the onset of reperfusion. The entire cooling procedure lasted for 3 hours before rewarming. Each group was further divided into 3 subgroups: histological observation at 24 h, and molecular analyses at 6 and 24 h after reperfusion, respectively. Observers were blinded to group assignments.

### Focal cerebral ischemia

Anesthesia was induced and maintained with 1.5-3.5% enflurane in 70% nitrous oxide and 30% oxygen. Rectal temperature was monitored during the procedure. Body temperature was kept at 36.5-37.5°C using a feedback-controlled heating blanket if needed. Blood gases and pressure were monitored via the right femoral artery which was cannulated with a PE-50 catheter. Rats were subjected to MCAO using the intraluminal filament. Briefly, a 4.0 nylon suture with a blunted tip coated with poly-L-lysine was inserted into the right external carotid artery and lodged in the narrow proximal anterior cerebral artery to block the MCA at its origin as described previously [16]. Two hours after occlusion, reperfusion was established by withdrawal of the filament under anesthesia.

### Physical hypothermia

Ice pads were placed under the ischemic rats as previously described by us [19]. Rectal temperature was reduced to and maintained at 31°C before rewarming.

### DHC treatment

DHC, at dose of 1.5 mg/kg (H-DHC) or 0.5 mg/kg (L-DHC), was administered via intraperitoneal (i.p.) injection. Body temperature was reduced to and maintained at 31°C by setting a heating pad at 31°C. For normal temperature low-dose DHC group, rectal temperature was maintained at 37.8-38.3°C by placing rats on heating pad.

### Combination therapy

DHC (0.5 mg/kg) was administered simultaneously with ice pad. Similarly, body temperature was reduced to and maintained at 31°C by a heating pad set at 31°C.

### Temperature maintenance and Rewarming

After 3 h of cooling procedure, lamp and heating pad were used in re-warming. The rewarming time was controlled in the same manner for each group.

### Neurological Deficits

Neurological deficits were evaluated at 24 h after reperfusion by 5- and 12-point neurological scale systems as described previously by us [16, 20, 21]. Higher scores indicate more severe deficits in both scoring systems.

### Cerebral infarct volume

Infarct volume was evaluated at 24 h after reperfusion as described previously by us [16, 21]. Six coronal brain slices with a 2-mm thickness were cut for 2,3,5-triphenyltetrazolium chloride (TTC, Sigma, St. Louis, MO, USA) staining at 37°C. In order to minimize error caused by edema, an indirect percentage was used to calculate final infarct volume [22].

### ROS Production

ROS generation was assessed with the Amplex Red Hydrogen Peroxide/Peroxidase Assay Kit (Thermo Fisher Scientific, Waltham, MA, USA) as previously described by us [23]. Homogenized brain samples were diluted to 10mg/ml based on protein concentration and 100 μg/ml of digitonin was added. After incubation for 30 min, the Amplex Red and Horse Radish Peroxidase were added, then H_2_O_2_ levels in brain homogenates were detected at 37°C on a Varioskan Flash Multimode Reader (Thermo Scientific, Waltham, MA, USA).

### ATP Assay

ATP level was measured with an ATP assay kit (ATP Colorimetric/FluorometricAssay Kit; BioVision, Milpitas, CA, USA). Per manufacturer protocol, brain tissue samples (10 mg) were homogenized with perchloric acid (PCA) and centrifuged at 13,000g for 2 min. Then the supernatant was mixed with ATP Assay Buffer, ATP probe, ATP Converter and Developer included in the kit. After a 30-min incubation avoiding light, a Varioskan Flash Multimode Reader (Thermo Scientific, Waltham, MA, USA) was used to quantify ATP levels (optical density (OD)=570 nm). Brain tissues were deproteinized by the Deproteinization Sample Preparation Kit (BioVision, Milpitas, CA, USA).

### Apoptotic Cell death

A cell death ELISA kit (Roche Diagnostics, Indianapolis, IN, USA) was used to quantify the degree of apoptosis in each group by measuring the amount of cytoplasmic histone-associated DNA fragments using a photometric enzyme immunoassay as described previously [15]. According to protocol, 10 mg brain sample was transferred to a 0.1 M citric acid solution, mixed with 0.5% Tween-20, then centrifuged at 2,000 rpms for 15min. The supernatant was diluted by incubation buffer and was used for assay. Absorbance at the 405-nm wavelength was detected for cell death using a Varioskan Flash Multimode Reader (Thermo Scientific, Waltham, MA, USA). Absolute values for cell death are presented.

### Protein expression

Western blot analysis was used to detect protein expression in the ischemic tissue, as described by us [24]. Membranes were incubated with primary antibodies including anti-cleaved Caspase-3 (1:200, Cell Signaling Technology), rabbit polyclonal anti-Bax (1:100, Santa Cruz; mouse monoclonal), rabbit polyclonal anti-Bcl-2 (1:100, Santa Cruz; mouse monoclonal), and anti-Bcl-xL (1:100, Santa Cruz; rabbit monoclonal), for 24h at 4^0^C. An ECL-system was used to detect immunoreactive bands by luminescence. Western blot images for each antibody, including β-actin, were analyzed using an image analysis program (ImageJ 1.42, National Institutes of Health, USA), to quantify protein expression in terms of relative image density. The mean amount of protein expression from the sham-operation group was assigned a value of 1 to serve as reference. Expression of target genes was represented as the fold-differences with respect to the control.

### Statistical Analysis

All numerical data were described as mean ± SEM. Statistical analysis was performed with SPSS for Windows, version 21.0 (IBM Corporation, Somers, NY, USA). The differences among groups were assessed using two-way analysis of variance with a significance level of *p*<0.05. Post-hoc comparison between groups was further detected using the least significant difference (LSD) method.

## RESULTS

### Physiological parameters

There were no significant differences in blood pH, PaCO_2_, PaO_2_, and MAP (mean arterial pressure) among all groups.

### Onset of hypothermia

**Figure 1:**
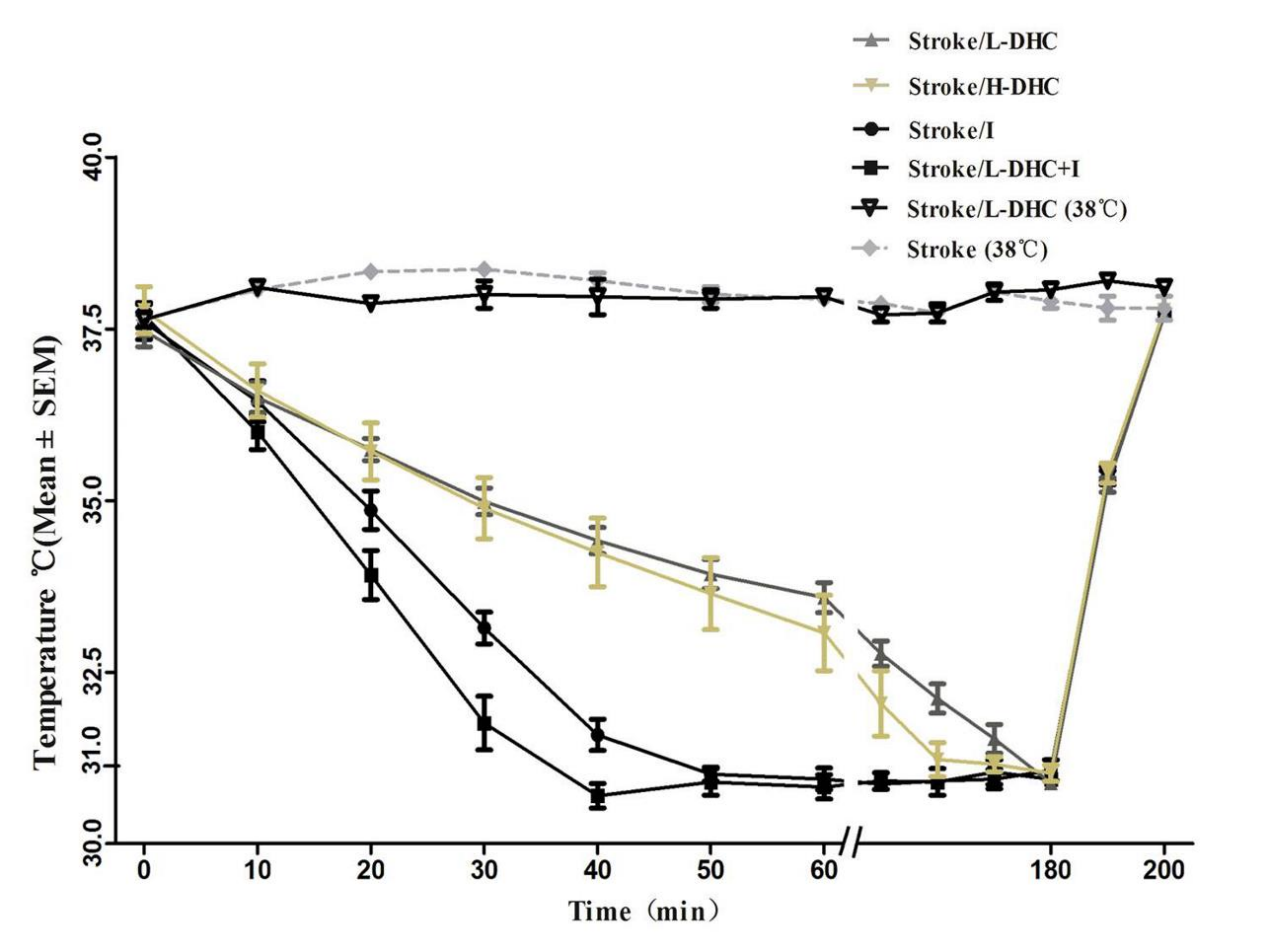
Changes of body temperature during hypothermia procedure in rat after MCAO The figure shows changes in rectal temperature after reperfusion (0-180 min) for different therapies in MCAO groups, including L-DHC (0.5 mg/kg), H-DHC (1.5 mg/kg), ice pad, L-DHC combined with ice pad, and L-DHC with normal temperature group. The baseline temperatures were measured immediately after reperfusion, and all therapies were initiated after recording the baseline temperatures. No statistical differences were observed in baseline temperatures, target temperatures or endpoint temperatures in all these groups. In the combination therapy group, rectal temperature was reduced from 37.7 ± 0.07 °C to 31 ± 0.15 °C in less than 40 minutes. Ice pad rapidly reduced temperature from 37.6 ± 0.10 °C to 31 ± 0.09 °C after 50 minutes. The time for low and high DHC monotherapies to reach 31? were 180 and 120 min, respectively.

Body temperature was maintained at 37.8-38.3°C in the control group as well as one L-DHC treatment group. Body temperatures in other groups receiving different treatments (L-DHC, H-DHC, ice pad (I) and L-DHC/I) were significantly reduced (Fig. 1) with different cooling rates. DHC treatments with either high or low dose slowly reduced body temperatures and reached the target of 31^0^C at 120 and 180 min, respectively. In contrast, target temperature in the combination (L-DHC/I) group was reached within 38 min as compared to 50 min by ice pad alone. The cooling rate induced by L-DHC/I (0.18 ± 0.03°C/min) was significantly (*F*_[3,28]_=106.7; *p*<0.001) faster than all other groups: ice pad by 28.6% (0.14 ± 0.01°C/min), H-DHC by 200% (0.06 ± 0.01°C/ min), and L-DHC by 350% (0.04 ± 0.01°C/min).

**Figure 2:**
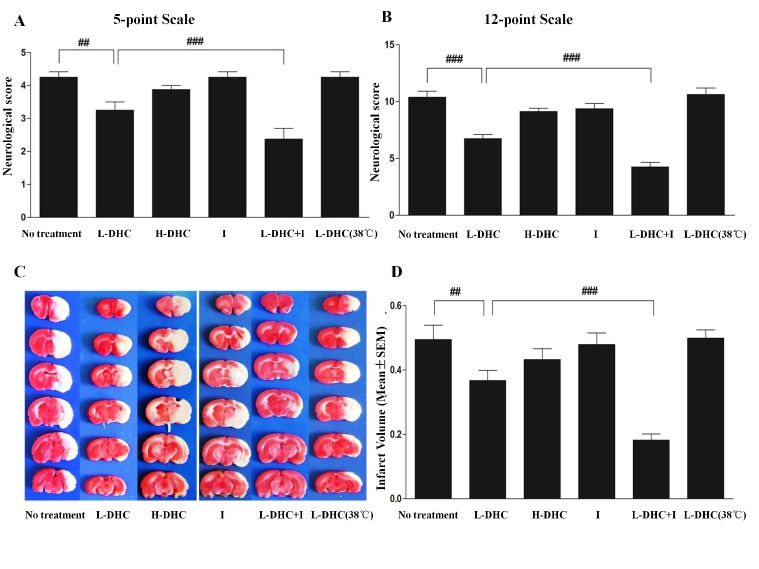
Neurological deficits and brain infarct volumes at 24 h of reperfusion Higher scores represent greater deficits in neurological function in the 5-point (**A**) and 12-point scale (**B**) systems after stroke. At 24 h after reperfusion, slightly lower scores (3.3 ± 0.7, 6.8 ± 0.5) were observed in L-DHC group than no treatment group (4.3 ± 0.3, 10.6 ± 0.4) (##p<0.01, ###p<0.001). In the L-DHC with normal temperature (4.3±0.4, 10.6 ± 0.3) (##p<0.01, ###p<0.001), these reductions were statistically reversed. The combination therapy largely reduced deficit score (2.4 ± 0.5, 4.3 ± 0.4) (###p<0.001, ###p<0.001) as compared with L-DHC and ice pad each alone. Neither ice pad (4.3 ± 0.3, 9.4 ± 0.3) nor H-DHC group (3.9±0.4, 9.1±0.5) showed significant therapeutic effects. (**C**) Representatives of brain slices with TTC staining after ischemia/reperfusion in each group are shown. (**D**) At 24 h of reperfusion, the stroke group without treatment and L-DHC with normal temperature (37°C) showed infarct volume of 49.5 ± 12.4% and 50.0 ± 11.2%, respectively. L-DHC significantly (##p<0.01) reduced infarct volume (36.7 ± 9.9%). The combination of L-DHC with ice pad further reduced infarct volume to 18.2 ± 5.8% (###p<0.001). Neither ice pad (47.9 ± 10.3%) nor H-DHC (1.5mg/kg) (43.3 ± 9.4%) alone showed a therapeutic effect.

### Neurological Deficit

Significant neurological deficits determined by 5 score (4.3 ± 1.1 out of 5 points, Fig. 2A) and 12 score (10.4 ± 0.8 out of 12 points, Fig. 2B) systems were measured at 24 h of reperfusion in ischemic rats. These deficits were reduced by 23.5% (*F*_ [2, 21]_ = 8.6; *p*<0.01) or 35.0% (*F*_ [2, 21]_ = 12.9; *p*<0.01) with L-DHC treatment, but not H-DHC (4.3 ± 0.1, 9.4 ± 0.4) and ice pad (4.3 ± 0.1, 9.4 ± 0.4). However, L-DHC combined with ice pad largely reduced the deficits by 44.0% (*F*_ [3, 28]_ = 19.2, *p*<0.001) with 5 scales or 59.1% (*F*_ [3, 28]_ = 38.1, *p*<0.001) with 12 scale system. L-DHC with temperature control at normal level (4.3 ± 0.1, 10.6 ± 0.4) did not differ from stroke group.

### Infarct Volume

Ischemia induced a 49.5 ± 12.4% infarct volume of cerebral hemisphere at 24 h of reperfusion. This infarction was reduced to 36.7 ± 9.9% by L-DHC (*F*_[2, 21]_ = 5.1; *p*<0.01; Fig. 2C and D), but not H-DHC (43.3 ± 9.4%) and ice pad (47.9 ±10.3%). When L-DHC was combined with ice pad, infarct volume was further reduced to 18.2 ± 5.8% (*F*_[3, 28]_ =18.1; *p*<0.01). However, L-DHC did not reduce the infarct volume (50.0 ± 11.2%), while body temperature was maintained at ~38°C.

**Figure 3:**
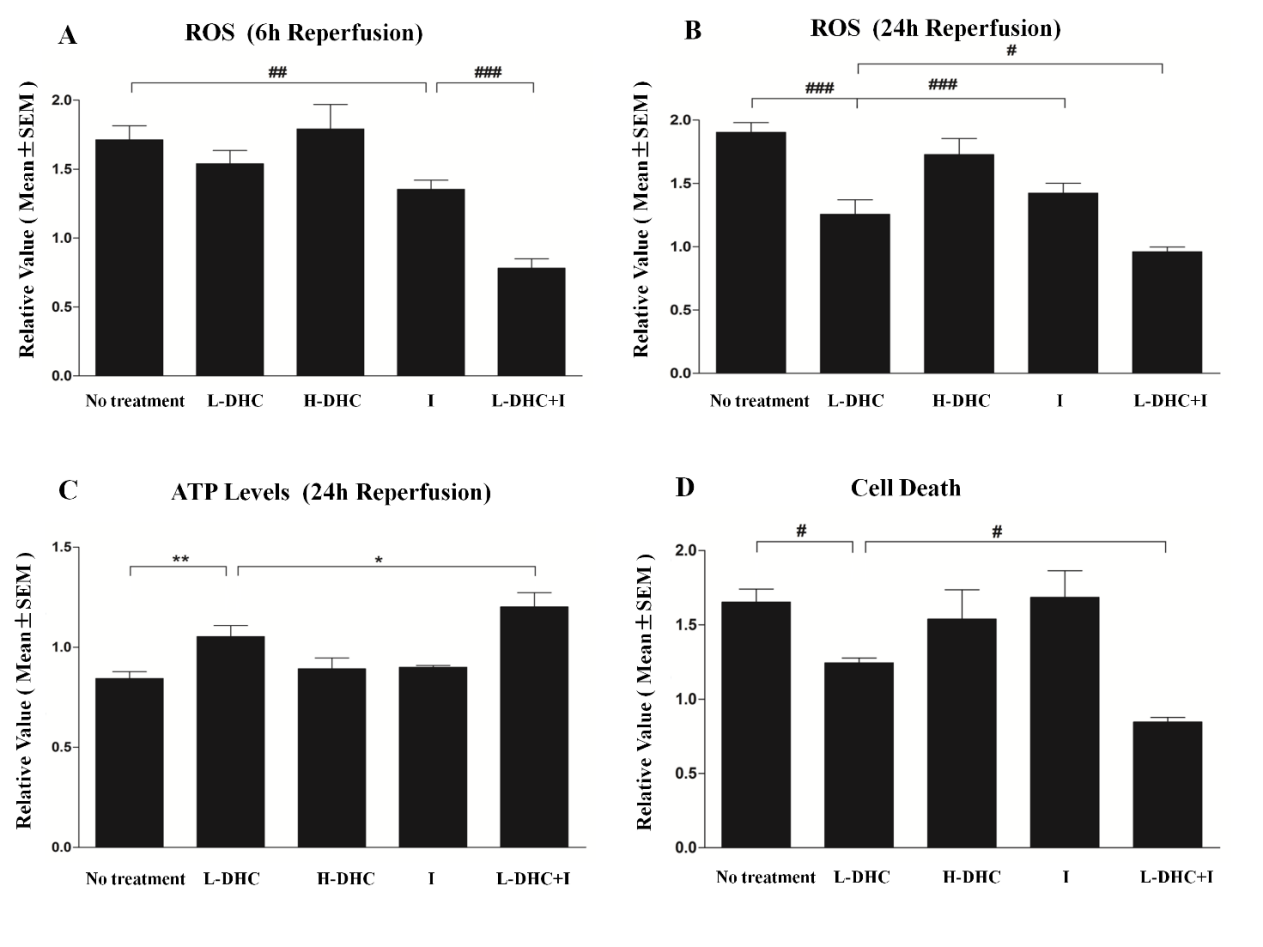
ROS production, ATP levels and cell death At 6 (**A**) and 24 h (**B**) after reperfusion, reactive oxygen species (ROS) levels were significantly elevated (***p<0.001, ***p<0.001) in no treatment group as compared to sham operation control group (reference as 1). Ice pad group significantly reduced ROS levels at 6 (##p<0.01) and 24 h (###p<0.001). L-DHC group significantly (###*p*<0.001) reduced ROS levels at only 24 h. Again, combination therapy largely enhanced the reduction in ROS levels at both time points (###p<0.001, #*p*<0.05). (**C**) ATP production was compared among treatment groups at 24 h reperfusion. MCAO reduced ATP levels significantly (#p<0.05) compared with sham operation group (artificially set to 1). L-DHC group significantly (**p<0.01) elevated ATP levels, and this was further enhanced by combination therapy (*p<0.05). Other therapies did not show significant changes in ATP. (**D**) Apoptotic cell death profile was compared among groups at 24 h reperfusion. The degree of apoptotic cell death was significantly (***p<0.001) increased in no treatment stroke group than sham operation group. A moderate reduction (#*p*<0.05) in cell death was observed in L-DHC group, and this reduction was enhanced by the combination therapy (#*p*<0.05). Other treatment groups did not show significant reduction in cell death.

**Figure 4:**
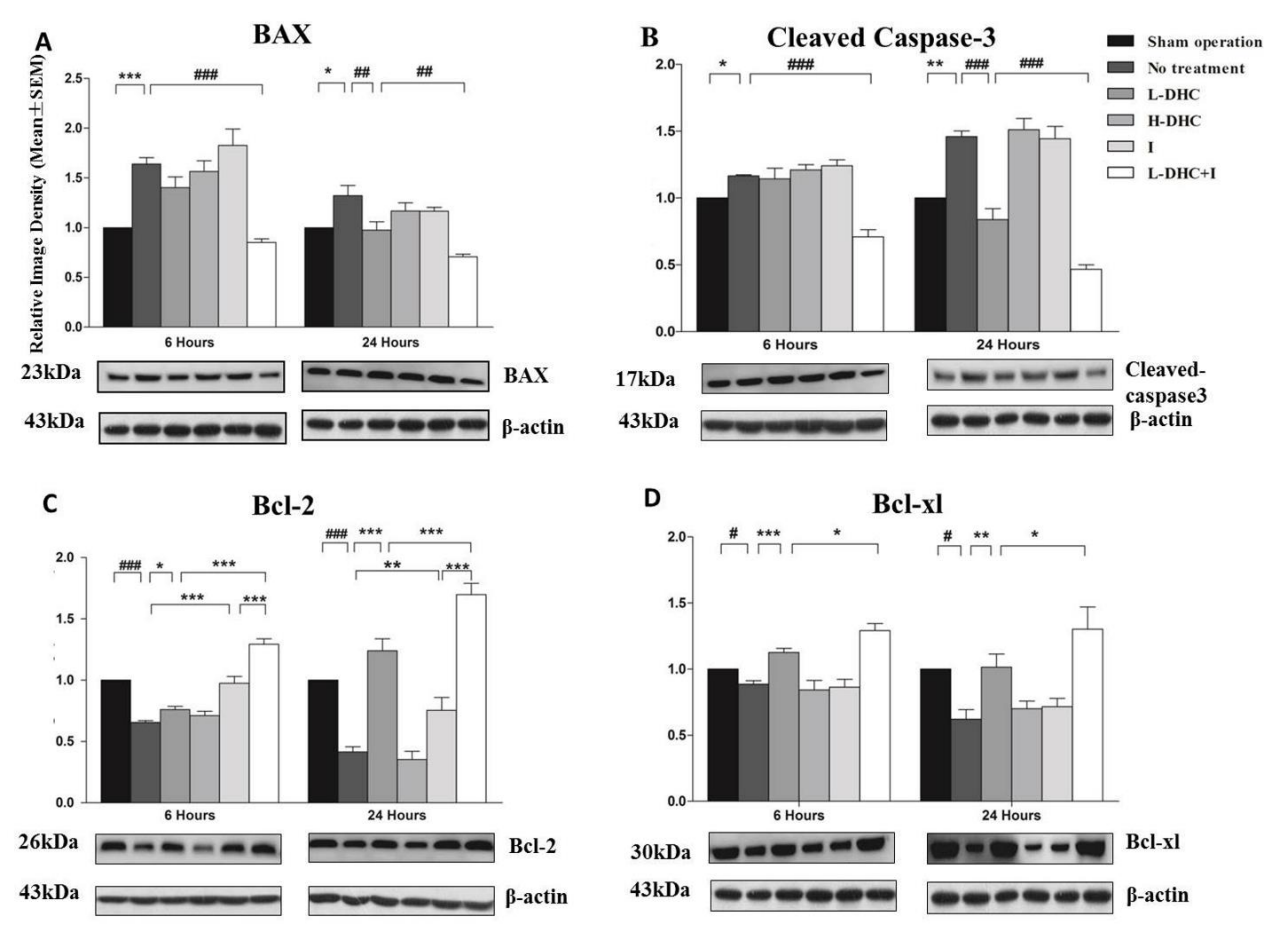
Apoptotic protein expression evaluated by Western blotting (**A**) Bax protein expression was significantly (***p<0.001, *p<0.05) increased in no treatment group at 6 (***p<0.001) and 24 h (*p<0.05) compared with sham control (artificially set at 1). At 6 h, the combination therapy significantly (###*p*<0.001) reduced protein expression. At 24 h, L-DHC group significantly (##*p*<0.01) reduced protein expression. This reduction was enhanced by combination therapy (##*p*<0.01). (**B**) Cleaved caspase-3 protein expression was significantly increased after stroke at 6 (*p<0.05) and 24 h (**p<0.01) of reperfusion. The combination therapy significantly (###*p*<0.001) reduced protein expression at 6 h of reperfusion. At 24 h, the reduced effect of L-DHC on cleaved caspase-3 protein expression (###*p*<0.001), was significantly (###*p*<0.001) enhanced by combination therapy. (**C**) Bcl-2 protein expression was significantly reduced after stroke at both 6 (###*p*<0.001) and 24 (###p<0.001) h of reperfusion. L-DHC and ice pad significantly increased the protein expression at 6 (*p<0.05, ***p<0.001) and 24 h (***p<0.001, **p<0.01) of reperfusion. The combination therapy augmented the increase seen in L-DHC (***p<0.001, ***p<0.001) and ice pad groups (***p<0.001, ***p<0.001) at both time-points. (**D**) Bcl-xl protein expression was significantly reduced after stroke at the two-time points after reperfusion (#*p*<0.05, #p<0.05). While L-DHC significant increased the protein expression at both time-points (***p<0.001, **p<0.01), the combination therapy enhanced the increase at 6 (*p<0.05) and 24 (*p<0.05) of reperfusion.

### ROS levels

Oxidative stress after stroke was significantly increased at 6 h (*F*_[5,42]_=19.0, *p*<0.001; Fig. 3A) and remained elevated at 24 h (*F*_[5,42]_=16.4, *p*<0.001; Fig. 3B) as compared to sham control referenced as 1. L-DHC reduced ROS levels by 34.4% at 24 h (*p*<0.001) but not 6 h, while H-DHC did not reduce ROS generation at both time points. Ice pad induced a milder reduction in ROS levels by 21.1% at 6 h (*p*<0.01) and 26% at 24 h (*p*<0.001). However, L-DHC and ice pad together greatly decreased ROS levels by 54.4% (*p*<0.001) or 50.0% (*p*<0.001) at 6 and 24 h, respectively.

### ATP levels

Brain metabolic activity was evaluated by ATP levels. When compared with sham-operated group referenced as 1, ATP level was significantly decreased (*F*_[5,42]_=9.3, *p*<0.001, Fig. 3C) at 24 h after reperfusion. L-DHC significantly (*p*<0.01) increased the ATP levels by 25%, while H-DHC and ice pad alone did not increase ATP levels. Combination of L-DHC and ice pad produced a larger increase by 42.9% (*p*<0.001).

### Apoptotic cell death

Level of apoptotic cell death was greatly increased in the stroke group compared to the sham-operated group (reference as 1) (*F*_[5,42]_=6.9, *p*<0.001, Fig. 3D). L-DHC decreased (*p*<0.01) the cell death by 24.9% while H-DHC and ice pad alone did not show neuroprotection. Again, combination of L-DHC and ice pad produced the greatest decrease in cell death by 48.5% (*p*<0.001).

### Pro-apoptotic protein expression

As compared to sham control (reference as 1), stroke significantly increased protein expression of Bax at 6 (*F*_[5,42]_=15.43; *p*<0.001) and 24 h (*F*_[5,42]_=10.3; *p*<0.001)(Fig. 4A). L-DHC decreased Bax expression (*p*<0.01) at 24 h. H-DHC and ice pad alone did not significantly alter the Bax expression as compared to stroke group. The greatest reduction in Bax expression was induced by the combination of L-DHC and ice pad at both time points (*p*<0.001).

### Cleaved caspase-3 expression

Cleaved caspase-3 is the active form of caspase-3. Stroke significantly increased cleaved Caspase-3 protein level as compared to sham control, reference as 1, at 6 (*F*_[5,42]_=18.6; *p*<0.001) and 24 h (*F*_[5,42]_=42.7; *p*<0.001)(Fig. 4B). L-DHC decreased the expression of cleaved Caspase-3 (*p*<0.001) at 24 h, while H-DHC and ice pad alone did not significantly alter the expression. The greatest reduction in cleaved Caspase-3 expression was induced by the combination of L-DHC and ice pad at both time points (*p*<0.001).

### Anti-apoptotic protein expression

When compared with sham-operated group (reference as 1), protein expression of Bcl-2 (Fig. 4C) and Bcl-xL (Fig. 4D) was markedly reduced by stroke at both 6 (*F*_[5,42]_=47.5, *p*<0.001; *F*_[5,42]_=16.2, *p*<0.05) and 24 h (*F*_[5,42]_=60.0, *p*<0.001; *F*_[5,42]_=8.3, *p*<0.05). At the two time-points, L-DHC moderately but significantly increased Bcl-2 and Bcl-xL expressions, while ice pad only affected Bcl-xL. The greatest increase of the two anti-apoptotic proteins was obtained by the combination at both time points (*p*<0.001).

## DISCUSSION

The present study revealed that combining pharmacological (0.5 mg/kg DHC) and physical (ice pad) hypothermia techniques synergistically induced superior neuroprotection to either approach alone. This is evidenced not only by significant reduction in infarct volumes and neurological deficits but also with improved overall cellular oxidative profile as well as reduction in apoptotic cell death. These findings support our hypothesis that combination treatment has potentially safer and more relevant clinical applications than monotherapies.

### Physical and Pharmacological Hypothermia

It has been established in previous literature that hypothermia has promising neuroprotective effects following ischemic stroke [25, 26] and the use of physical TH has been investigated in a number of clinical trials [27]. Several mechanisms have been proposed to explain this effect including its ability to reduce oxygen demand while preserving energy stores, decrease metabolic rate and suppress cell death [28]. However, outcomes of this therapy are largely dependent on multiple factors such as time to initiation of therapy, duration of therapy as well as the depth of hypothermia [6]. It has been shown that lower temperatures that are achieved rapidly and maintained for long periods have potentially better outcomes [26, 28, 29]. However, determining the optimal depth and duration of hypothermia is still the subject of much debate and is complicated by the fact that those preferred parameters are associated with higher chances of side effects. Additionally, the depth of physical TH is limited by physiological thermoregulatory responses, such as shivering. These responses hinder efforts to cool the body and may lead to prolonged induction of hypothermia and less stable maintenance. All of these factors collectively limit the scope of TH’s clinical use and necessitates further research. To counteract those limitations, we developed a combination therapy using physical and pharmacological hypothermia and were able to show its promising effects on a wide array of parameters.

Transient Receptor Potential Vanilloid channel 1 (TRPV1) is a nonspecific cation channel that is highly expressed in warm-sensing nerve fibers in peripheral and central nervous system [6]. It is believed that TRPV1 plays a role in thermoregulation by reducing the thermoregulatory set-point at peripheral thermosensors and pre-optic anterior hypothalamus POAH [30]. TRPV receptors have been shown to be activated by capsaicin (an active component of chili peppers), DHC (a saturated structural analog of capsaicin), and rinvanil (a capsaicin analogue) in animal models [12, 31, 32]. Particularly, DHC was used as a potential neuroprotective agent given its ability to induce systemic hypothermia of 33.0± 0.2 ˚C after 100 minutes in a rodent stroke model [33]. However, over activation of TRPV1 by these agonists was reported to have deleterious effects on brain pathophysiology by a number of mechanisms [34]. Stimulating TRPV1 channel increases the frequency of spontaneous excitatory postsynaptic currents in dopaminergic neurons and promotes glutamate release [35-37]. This subsequently leads to excitotoxicity in the CNS, which is a key mechanism for the degeneration of neurons after ischemia [38]. Additionally, TRPV1 endogenous agonists that are elevated in ischemic environment [38, 39] lead to neurotoxicity through Ca^2+^ influx, ERK activation, and ROS generation [40]. Taken together, over stimulation of TRPV1 receptors results in more deleterious effects than its potentially neuroprotective ones and that narrows its clinical potential. To overcome this limitation, we used the TRPV1 agonis with physical hypothermia to achieve neuroprotection safely and effectively.

### Synergistically Induced Hypothermia

Although temperature regulation is a complex physiological process controlled centrally by Pre-Optic Anterior Hypothalamus (POAH) and peripherally by thermo-sensors [40], our combination approach was able to stimulate both arms by targeting internal thermoregulation using DHC and peripheral heat loss using ice pads. This novel approach not only achieved hypothermia more rapidly but also resulted in more enhanced neuroprotection than previous studies [12, 33]. The combination of DHC and ice pads shortened the duration to reach the target temperature of 31°C by more than 12 minutes as compared to physical hypothermia alone, and by 80 minutes as compared to DHC monotherapy. When body temperature was controlled at normal level, the use of DHC (0.5 mg/kg) did not induce neuroprotection. This finding confirms the previously established mechanism for DHC’s neuroprotection effects by its ability to induce hypothermia [33]. High-dose DHC did not provide neuroprotective effects in this study. This could be explained by the neurotoxic effects resulting from excessive TRPV1 agonism, as mentioned above [41]. Furthermore, physical TH, induced by ice pad, did not provide consistent neuroprotection in our study. This observation can be due to the fact that although physical TH is effective in reducing surface temperature, its effect on the central thermoregulation is drastically lower, leading to greater physiological thermoregulatory responses and thus unstable hypothermia.

### Neuroprotective Mechanisms

Different mechanisms explain physical TH’s induced neuroprotection including the down-regulation of energy metabolism and improvement of oxidative phosphorylation [9, 10, 42] which was proven in this study. Our therapy significantly increased ATP production which leads to a more favorable cellular metabolism and mitochondrial functional status. The reduction in ATP levels contributes to the cellular swelling and imbalanced intracellular ion gradient that is seen following an ischemic injury.

ROS play a significant role in brain injury after stroke [43]. It is produced by oxidative phosphorylation dysfunction, mitochondrial injury, and reperfusion injury [25, 43, 44]. Our results confirm the effect of reduced ROS production by TH, leading to reduced ischemic injury. On the other hand, high-dose DHC did not show significant decrease in ROS, possibly from excessive TRPV1 activation resulting in increased ROS generation as mentioned above [41]. At 24 h of reperfusion, the reduced ROS levels were found to be associated with reduced brain infarct volume and neurological deficits, suggesting that at least the neuroprotection was partly due to the reduced ROS. We will further investigate this relationship in subsequent studies.

Following ischemic injury, apoptosis is mediated by a number of signaling pathways through a very complex mechanism. The intrinsic pathway of apoptosis is regulated by mitochondrial associated B-cell lymphoma 2 (Bcl-2) protein family. These include both pro-apoptotic proteins such as Bcl-2-associated X (Bax), Bad, and Bak and anti-apoptotic proteins, such as Bcl-2 and Bcl-xl [45]. Studies demonstrated that decrease in Bax-Bad/Bcl-2-Bcl-xl ratios plays a significant role in protective preconditioning against lethal ischemic injury [46]. Bcl-2 proteins are also regulators in the process of cytochrome c release and caspase activation. Caspase-3 is an aspartate-specific cysteine protease which is regarded as an executioner caspase involved in carrying out degradative functions inside the cell, such as protein and DNA degradation [47, 48]. Caspase-3 is activated to cleaved caspase-3 in apotosis [8]. Our combined therapy corrected apoptotic protein levels by down-regulating pro-apoptotic factors Bax while simultaneously increasing pro-survival proteins Bcl-2 and Bcl-xl. This sheds lighter on elucidating the protective mechanism of TH and further proves previous research [49].

Ischemic stroke has a multitude of catastrophic consequences leading to a large number of cellular pathways going awry. There is a lack of neuroprotective therapies that can target this complex cascade of events to salvage injured neurons following stroke. In comparison with a very recent study on the benefit of combining physical cooling with pharmacological hypothermia in stroke [50], in our study, stroke was more severe with longer MCAO and the pharmacological method was different. Specifically, we used a very low dose of TRPV1 agonist DHC (0.5 vs. 1.5mg/kg) to enhance the effect of systemic cooling, in order to achieve faster hypothermia and stronger neuroprotection. Importantly, our study established a concept of synergistic therapeutic hypothermia by using both ice pad and low dose DHC cooling approaches. Our results highlighted the fact that combining physical and pharmacological hypothermia is a promising therapy to improve outcome in ischemic stroke. We also investigated multiple cellular mechanisms underlying the observed neuroprotection, moving hypothermia a step closer to be implemented as an effective treatment. In the present study, rewarming time was about 20 min. We will determine the effect of different rewarming time on outcomes in future studies. The anti-inflammatory effect is another important mechanism underlying TH-induced brain protection [51]. Thus, further research is needed to understand those effects and evaluate the long-term effectiveness of our therapy.
